# Antibacterial Biodegradable Films Based on Alginate with Silver Nanoparticles and Lemongrass Essential Oil–Innovative Packaging for Cheese

**DOI:** 10.3390/nano11092377

**Published:** 2021-09-13

**Authors:** Ludmila Motelica, Denisa Ficai, Ovidiu-Cristian Oprea, Anton Ficai, Vladimir-Lucian Ene, Bogdan-Stefan Vasile, Ecaterina Andronescu, Alina-Maria Holban

**Affiliations:** 1 Faculty of Applied Chemistry and Material Science, University Politehnica of Bucharest, 060042 Bucharest, Romania; motelica_ludmila@yahoo.com (L.M.); denisa.ficai@upb.ro (D.F.); anton.ficai@upb.ro (A.F.); vladimir.l.ene@gmail.com (V.-L.E.); vasile_bogdan_stefan@yahoo.com (B.-S.V.); ecaterina.andronescu@upb.ro (E.A.); alina.m.holban@bio.unibuc.ro (A.-M.H.); 2 Academy of Romanian Scientists, 050045 Bucharest, Romania; 3 Microbiology & Immunology Department, Faculty of Biology, University of Bucharest, 077206 Bucharest, Romania

**Keywords:** biodegradable, alginate film, antibacterial packaging, lemongrass essential oil, silver nanoparticles, edible packaging, cheese, time-temperature indicator (TTI)

## Abstract

Replacing the petroleum-based materials in the food industry is one of the main objectives of the scientists and decision makers worldwide. Biodegradable packaging will help diminish the environmental impact of human activity. Improving such biodegradable packaging materials by adding antimicrobial activity will not only extend the shelf life of foodstuff, but will also eliminate some health hazards associated with food borne diseases, and by diminishing the food spoilage will decrease the food waste. The objective of this research was to obtain innovative antibacterial films based on a biodegradable polymer, namely alginate. Films were characterized by environmental scanning electron microscopy (ESEM), Fourier-transform infrared spectroscopy (FTIR) and microscopy, complex thermal analysis (TG-DSC-FTIR), UV-Vis and fluorescence spectroscopy. Water vapor permeability and swelling behavior were also determined. As antimicrobial agents, we used silver spherical nanoparticles (Ag NPs) and lemongrass essential oil (LGO), which were found to act in a synergic way. The obtained films exhibited strong antibacterial activity against tested strains, two Gram-positive (*Bacillus cereus* and *Staphylococcus aureus*) and two Gram-negative (*Escherichia coli* and *Salmonella* Typhi). Best results were obtained against *Bacillus cereus*. The tests indicate that the antimicrobial films can be used as packaging, preserving the color, surface texture, and softness of cheese for 14 days. At the same time, the color of the films changed (darkened) as a function of temperature and light presence, a feature that can be used to monitor the storage conditions for sensitive food.

## 1. Introduction

The majority of food packaging materials used at present are based on petrochemical products or cellulose, due to historical factors such as low cost or mechanical and barrier properties [1,2]. The pressure of environmental concerns will phase out the petroleum-based materials, which will increase the need for innovative, biodegradable polymeric packaging materials such as chitosan [3], alginate [4], cellulose [5], starch [6], pullulan [7], polylactic acid [8], etc. The need to decrease the food waste, and the desire to increase the food safety and to prolong the shelf life creates pressure on the food packaging industry to develop and adopt new antimicrobial materials [9,10,11]. Besides chitosan, none of these biopolymers present antimicrobial activity [12]. Therefore, various antimicrobial agents have to be mixed with the polymeric matrix to obtain the desired antibacterial or antifungal activities [13]. Such innovative antimicrobial biodegradable materials can diminish the microorganisms’ proliferation and thus will reduce the food spoilage, increase the shelf life, and help provide a better food quality [14,15,16,17,18].

Sodium alginate (A) belongs to the polysaccharides class, being the salt of alginic acid [19]. Usually, the source of alginate is the marine algae brown seaweed, which makes it acceptable for people with religious dietary restrictions [20]. Alginate is one of the most versatile polymers, being used in various applications from drug delivery systems [21], to environmental depollution [22], wound healing, and tissue engineering [23]. Alginate is considered GRAS (generally recognized as safe) by the US Food and Drug Administration, therefore is a natural choice for packaging materials [24,25]. It is water-soluble and can be easy functionalized [26]. Mechanical properties are enhanced by adding plasticizers, glycerol being the most common choice due to its superior compatibility with the polymeric matrix [27,28]. Antibacterial activity can be bestowed to the alginate by adding various nanoparticles such as ZnO [29], Ag [30], CuO [31], natural extracts [32,33,34], or other substances of pharmaceutical interest [35,36,37].

Silver nanoparticles (Ag NPs) are one of the most potent antimicrobial agents [38,39,40]. The literature reports at least 650 microorganisms, including viruses, along with bacteria and fungi, affected by Ag NPs [41,42,43]. The shape and size of Ag NPs are the main factors that influence the antimicrobial activity, the smaller, triangular nanoparticles being more potent [39].

Essential oils and other natural plant extracts present a huge potential as antioxidants, antimicrobials, and even as insect repellants [44,45,46,47]. Lemongrass essential oil (LGO) is considered a bio-pesticide in the US [48]. The major constituents are citronellal, geraniol, and citronellol [49]. As a hydrophobic extract, the LGO addition to the alginate film will also improve the water vapor permeability (WVP) performance of the polymeric matrix [50,51,52].

For the LGO, there is no reported toxicity at concentrations used, but concerns are expressed about the impact on beneficial gut microbiota [53,54]. The introduction of nanomaterials such as Ag NPs into food packaging presents some potential drawbacks, beside advantages such as antimicrobial activity or improved UV-barrier properties. It is mandatory to study the toxicity related to the use of metallic nanoparticles. One way to minimize the impact is to decrease the concentration level of Ag NPs in the packaging films. By combining Ag NPs antimicrobial activity with that of LGO, we demonstrate that very low concentrations of metallic nanoparticles can be used, while still maintaining a strong antibacterial activity.

The objective of this research was to obtain a biodegradable, antibacterial material that can be used as packaging for soft cheese (usually with a shelf life of 4 days at 4–8 °C). The literature presents a couple of alginate-base films, with Ag NPs, used as packaging for meat [55,56] or vegetables [57], but none for cheese. In addition, to the best of our knowledge, here we report for the first time the obtaining of the alginate–Ag NPs–LGO system. We used two antimicrobials, Ag NPs and LGO, in order to enhance the final antibacterial activity of films, while introducing smaller amounts of antibacterial agents. The films were characterized from physico-chemical point of view, and the antibacterial activity was determined against two Gram-positive (*Bacillus cereus* and *Staphylococcus aureus*) and two Gram-negative (*Escherichia coli* and *Salmonella* Typhi) bacterial strains. The performed antibacterial test indicates that, while the film with the highest Ag NPs concentration exhibited the best antibacterial activity, the rest of films, with lower concentrations of Ag NPs, were still performing very well against tested bacterial strains. Therefore, combining the antimicrobial agents can be a successful strategy to decrease the amount of used substances, which limits their potential toxic activity against human organism.

## 2. Materials and Methods

### 2.1. Materials

Silver nitrate, polyvinylpyrrolidone (PVP), and sodium citrate were obtained from Merck. Sodium alginate (CAS 9005-38-3) was purchased from Fisher Scientific U.K. Ltd. (Redox Lab Supplies, Bucharest, Romania). Phosphate-buffered saline (PBS), sodium citrate, glycerol, nutrient broth, and agar were obtained from Sigma Aldrich (Redox Lab Supplies, Bucharest, Romania). Lemongrass essential oil (LGO) was purchased from Carl Roth (Amex-Lab, Bucharest, Romania). All the chemicals were used without any further purification.

The soft telemea cheese (S.C. Fabrica de lapte Brasov S.A., Halchiu, BV, Romania) was obtained from a local supermarket in Bucharest, Romania.

### 2.2. Synthesis of Silver Nanoparticles

AgNPs were synthetized as described in [58]. Briefly, 0.02 g AgNO_3_ was dissolved in 100 mL water under vigorous stirring at 70 °C. Then, 20 mL solution of 0.35 g sodium citrate was added dropwise as a reduction agent. After 30 min, a third solution (0.1 g PVP in 5 mL) was added dropwise. The yellow solution containing AgNPs (100 ppm) was used without further purifications.

### 2.3. Synthesis of Alginate/Ag/LGO Films

A certain amount of solution containing Ag NPs was mixed with 1 mL LGO and was sonicated further for 15 min before being used to prepare AAg1–AAg4 films (Table 1).

Alginate films were obtained by casting method. Shortly, 3 g alginate was added to a beaker of 100 mL water and left to dissolve for 24 h under stirring. Afterwards, 2 mL of glycerol was added to the alginate solution. A previously prepared emulsion of Ag NPs and LGO was added to the alginate solution, under vigorous stirring.

Each solution was put in a Petri dish and was left to dry in an oven for 24 h at 40 °C. A control film without Ag NPs and LGO was prepared in the same way. After drying, 200 mL CaCl_2_ solution (2%) was added to each Petri dish, and the films were left submerged for 10 min. Finally, the films were removed from the Petri dish and were stored in zip lock plastic bags at 20 °C and 60% relative humidity (RH) (Figure S1).

### 2.4. Characterization of Alginate Films

In order to investigate the films surface morphology and microstructure, scanning electron micrographs were obtained using an environmental scanning electron microscope VERSA 3D (ESEM, Thermo-Fisher, former FEI Company, Eindhoven, The Netherlands).

Bright Field and High Resolution a Transmission Electron Microscopy (BF-TEM, HR-TEM) images coupled with Selected Area Electron Diffraction (SAED) pattern were recorded using High-Resolution 80-200 TITAN THEMIS transmission microscope FEI (Thermo Fisher Scientific, Waltham, MA, USA).

Fourier transform infrared spectra were recorded with a Nicolet iS50 FTIR spectrometer (Thermo Fisher Scientific Inc., Waltham, MA, USA).

FTIR 2D maps were recorded with a Nicolet iS50R FTIR microscope (Thermo Fisher Scientific Inc., Waltham, MA, USA).

A Perkin Elmer (Waltham, MA, USA) LS55 spectrometer was used to measure the photoluminescence spectrum (PL).

A JASCO V560 spectrophotometer (JASCO Inc., Easton, PA, USA) was used to measure the UV–Vis spectra. The opacity values were calculated as A_600_/*x* = −logT_600_/*x*, where A_600_ is the absorbance at 600 nm, T_600_ is the fractional transmittance at 600 nm and *x* is the film thickness in mm. A higher opacity value indicates that the film is less transparent [59].

Thermal analysis, TG-DSC (thermogravimetry and differential scanning calorimetry), was performed with a STA 449C F3 apparatus, from Netzsch (Selb, Germany). The evolved gases were analyzed with a FTIR Tensor 27 from Bruker (Bruker Co., Ettlingen, Germany), equipped with a thermostated gas cell.

For the determination of water vapor permeability (WVP), we used permeation cups with a diameter of 30 mm, sealed with a sample film, as described in [60]. In each cup we placed 1 g of dried CaCl_2_. The permeation cups were placed in a container at a temperature of 25 °C and 100% relative humidity. Their weight was measured at fix intervals (8 h) for four days.

The swelling capacity was determined as described in [61]. Shortly, square samples of ~3 × 3 cm were cut from the fresh films and were dried in a desiccator for 48 h. Once dried, the samples were weighed (0.2 mg) (W_0_), then placed in 200 mL water or phosphate buffer saline (PBS) to allow swelling. The samples were first weighed at 15 min intervals, then each 30 min for three hours, and finally at 24 h intervals for the next days as the maximum swelling capacities were attained. The Equation (1) formula for degree of swelling (D) was used to calculate the swelling ratio:

D = (W_t_ − W_0_)/W_0_ (1)

The antibacterial activity was evaluated against two model Gram-positive (*Bacillus cereus* ATCC 13061 and *Staphylococcus aureus* ATCC 25923) and two Gram-negative (*Escherichia coli* ATCC 25922 and *Salmonella enterica* Typhi ATCC 14023) bacteria, which are relevant in food bacterial contamination. The strains were maintained as glycerol stocks at −80 °C. All experiments were designed and performed in triplicate.

To qualitatively screen the antibacterial effect of the obtained materials, we utilized an adapted diffusion assay, respecting the general rules exposed in the CLSI 2020 and in our recent study [62].

Cheese samples (~cubic shape with size of 2–3 cm) were packed in alginate and AAg1–AAg4 films and placed in a refrigerator (4 °C ± 1 °C and 75% R.H.) for 14 days. Samples were weighed again after 14 days for the mass loss test. Weight loss was monitored by measuring the mass change of each sample, and was calculated as percentage lost from the initial mass. The pH was measured initially and after 14 days.

The results were statistically evaluated using the analysis of variance (ANOVA) performed with Microsoft Excel 2016 (Microsoft Corp., Redmond, WA, USA), having installed the XLSTAT 2020.5.1 add-on. The Shapiro–Wilk test was used to check the normal distribution of the data; we assessed the homoscedasticity of the residuals by Levene’s test; and the results were compared by Tukey’s (HSD) test so that the pairs of films that differed in terms of statistical significance were revealed (*p* < 0.05).

More details about specific conditions for each analysis are presented in the Supplementary Materials.

## 3. Results and Discussion

The samples were characterized by transmission electron microscopy (TEM) and scanning electron microscopy (SEM), Fourier Transform Infrared (FTIR) spectroscopy and microscopy, UV-Vis and fluorescence spectroscopy, and thermal analysis TG-DSC. Water vapor permeability (WVP) and swelling behavior were also determined.

### 3.1. Transmision Electron Microscopy

**Figure 1:**
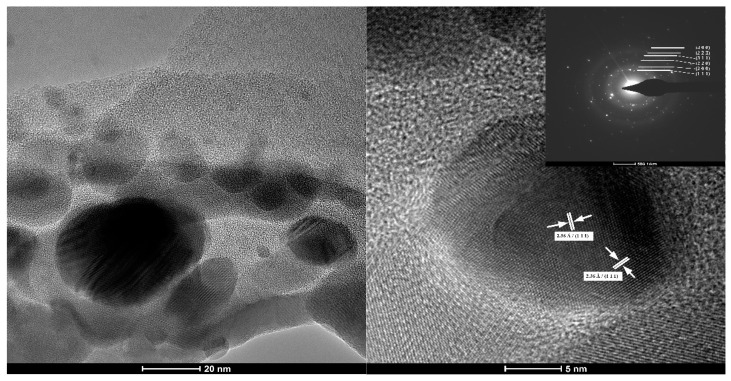
The TEM images for the obtained Ag NPs. Selected area electron diffraction (SAED) pattern in the inset.

The BF-TEM image presented in Figure 1 presents particles existing in the Ag NPs 100 ppm solution. They are round shaped, with a diameter of approximately 5 to 25 nm, having a bimodal distribution. The Ag NPs are highly crystalline, with bigger ones composed of 2–3 crystallites. Therefore, the large nanoparticles can be considered polycrystalline, while the smaller ones are monocrystalline. Similar distribution was reported before, for both laboratory synthesized and commercially available [63,64]. Most probably during grain formation, before adding the capping agent, some crystallites become agglomerate, thus larger nanoparticles grow along smaller ones.

The identified Miller indices in HR-TEM are the (111) corresponding to 2.36 Å distance. From SAED pattern, the only phase identified is that of FCC (face-centered cubic) silver, corresponding to the standard data JCPDS File No. 04-0783.

### 3.2. Environmental Scanning Electron Microscopy

**Figure 2:**
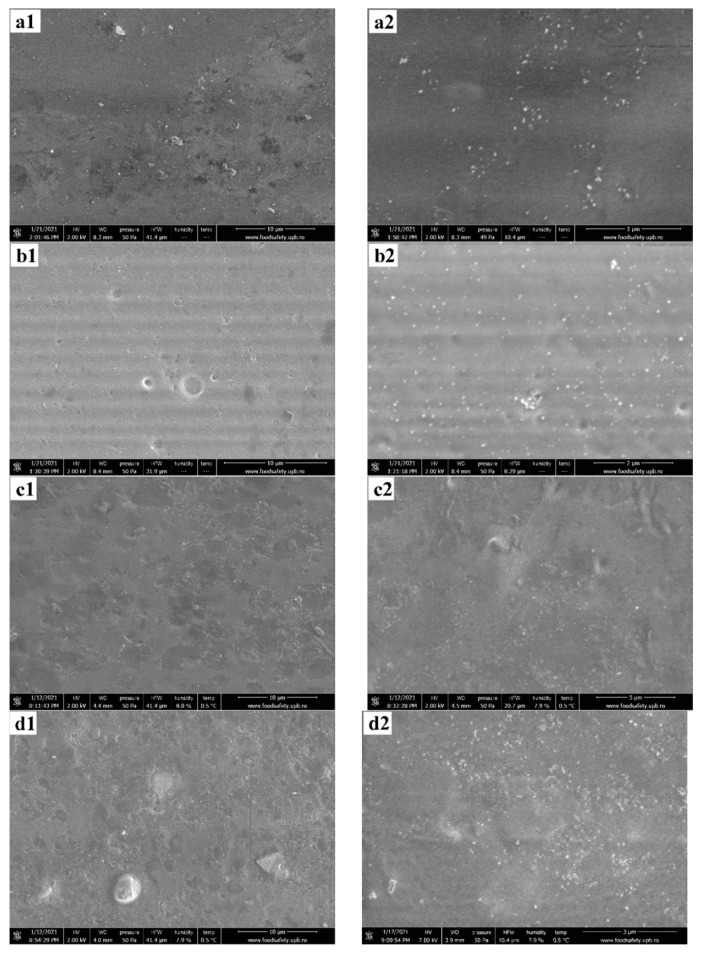
The ESEM micrographs for AAg1 (**a1**,**a2**); AAg2 (**b1**,**b2**); AAg3 (**c1**,**c2**); and AAg4 (**d1**,**d2**) films.

According to the pressure–humidity–temperature diagram of the liquid–vapor phase equilibrium for water, the expected pressure in the working chamber at a temperature of 0.5 °C and humidity of ~8% is estimated at ~50 Pa [65]. Thus, the analysis chamber was prepared by performing vacuum purge cycles, between pressures of 30–150 Pa, to avoid contamination of samples with impurities from the atmosphere. In order to capture the microstructure of the specimen, without the detrimental interference of high-vacuum and high-voltage, the image acquisitions are made in low vacuum conditions (50 Pa), a temperature of 0.5 °C, a relative humidity of ~8%, at a beam voltage of 2 kV, with a working distance of about 8.3 mm. The surface of the films is presented in Figure 2.

The ESEM images show that with the increasing content of Ag from AAg1 to AAg4, the surface of the alginate films does not change in either morphology nor pore size distribution. The only noticeable difference resides in the fact that Ag aggregates are more disperse in the specimens that contain a higher amount of nanoparticles, without significantly affecting their size. This observation leads to the idea that during the nucleation and crystal growth of silver nanoparticles, the PVP surfactant is limiting the crystal growth and stabilizing the aggregate size. The alginate polymer in which the Ag NPs were dispersed does not contribute towards further noteworthy agglomeration of nanoparticles due to sterically stabilization of Ag NPs from the synthesis stage [66].

**Figure 3:**
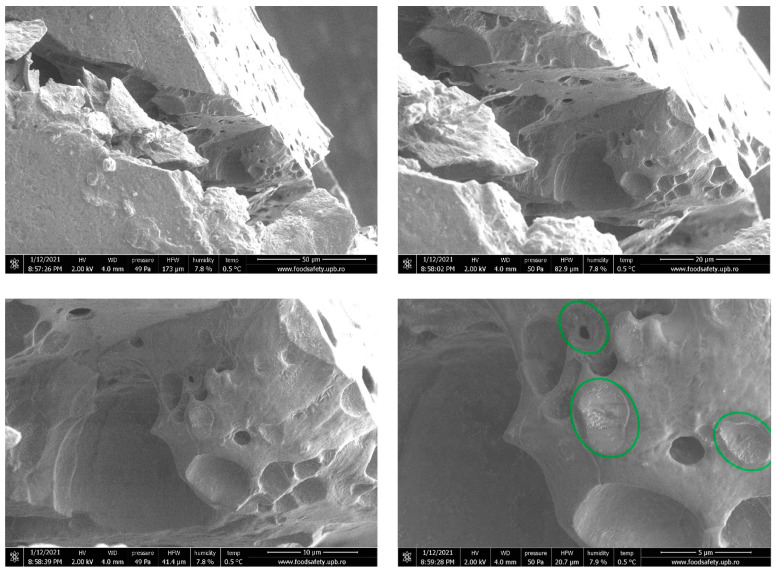
The near-fracture micrographs of AAg4 film. Ag NPs agglomerates inside pores.

More insightful information was extracted from a film fracture, presented in Figure 3. The interior of the polymeric film shows spherical micropores of variable sizes. Most probably, their formation is related to the presence of LGO microdroplets dispersion into the alginate matrix. Moreover, inside the polymer pores, Ag NPs agglomerates are found at the surface, suggesting that there is a positioning preference of the agglomerates at the pore-material interface, rather than a homogenous distribution of the latter throughout the material volume.

The presence of such micropores will most probably have an impact on mechanical and barrier properties of the films. Although the extent at which the overall mechanical and barrier properties of the film are affected by the preferred positioning of Ag NPs agglomerates on the pore-material interface is questionable, pores themselves act as points of minimal resistance for the film.

### 3.3. FTIR Spectroscopy and Microscopy

#### 3.3.1. FTIR Spectroscopy

The effects induced by the Ag NPs and LGO addition to the alginate matrix were investigated by FTIR spectroscopy. The peaks corresponding to the main absorption peaks and the associated vibration modes are presented in Table 2. The broad band from the 3270–3287 cm^−1^ corresponds to the presence of -O-H moiety from alginate and moisture as well.

The intense 2921–2930 cm^−1^ peaks are attributed to the C_sp3_-H symmetric vibrations of the alginate, while small 3075 cm^−1^ peak belongs to C_sp2_-H symmetric vibration from LGO components. The 1027–1031 cm^−1^ peaks were attributed to the glycosidic bond in the polysaccharide chain. The observed small shifts could indicate interactions between alginate negative charged -COO^-^ groups and uncoated zones of Ag NPs surface or the interaction of Ag NPs with -C = O moiety of the pyrrolidone cycle from the PVP coating [68].

#### 3.3.2. FTIR Microscopy

**Figure 4:**
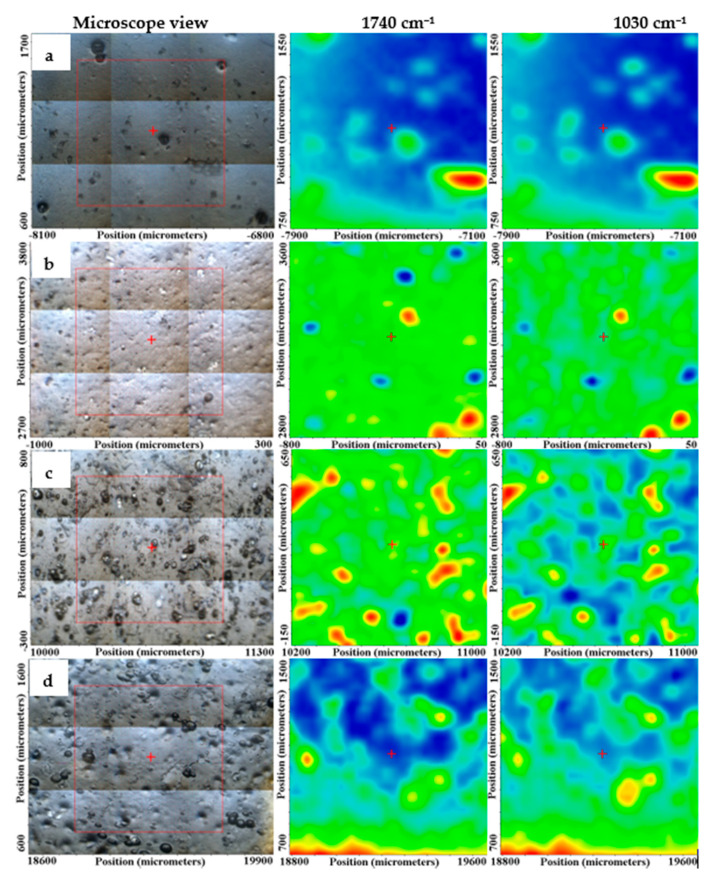
FTIR maps for the AAg1 (**a**); AAg2 (**b**); AAg3 (**c**); and AAg4 (**d**) films.

With the help of FTIR microscopy, we looked at spatial distribution of Ag NPs and LGO into the alginate matrix. The maps corresponding to the 1740 cm^−1^ and 1030 cm^−1^ are presented for the AAg1–AAg4 films in Figure 4, together with the microscopic view of the analyzed region.

The FTIR maps at those two wavenumbers for AAg1 and AAg2 are quite similar, indicating a uniform distribution of the components in the analyzed area. The increase in Ag NPs quantity in AAg3 and AAg4 films generated some local accumulations, clusters, or defects such as pores, which induce a less homogeneous structure for these two samples, at a level of tens of µm, maximum. Nevertheless, even for these two samples, the FTIR maps are not so different, therefore the samples can be considered as having a good homogeneity.

### 3.4. UV-Vis and Fluorescence Spectroscopy

#### UV-Vis Spectroscopy

The UV-Vis spectroscopy is widely used for the Ag NPs characterization and synthesis monitoring as it is sensible to the presence of nanoparticles and gives information about their morphology and size uniformity. The absorption peak generated by the localized surface plasmon resonance is the main characteristic of the UV-Vis spectrum for Ag NPs [69]. It can be observed at wavelengths starting from 400 nm up to 600 nm depending on Ag NPs shape and size [70,71,72]. In addition, UV-Vis spectra can be used to monitor the stability of the Ag NPs suspension.

**Figure 5:**
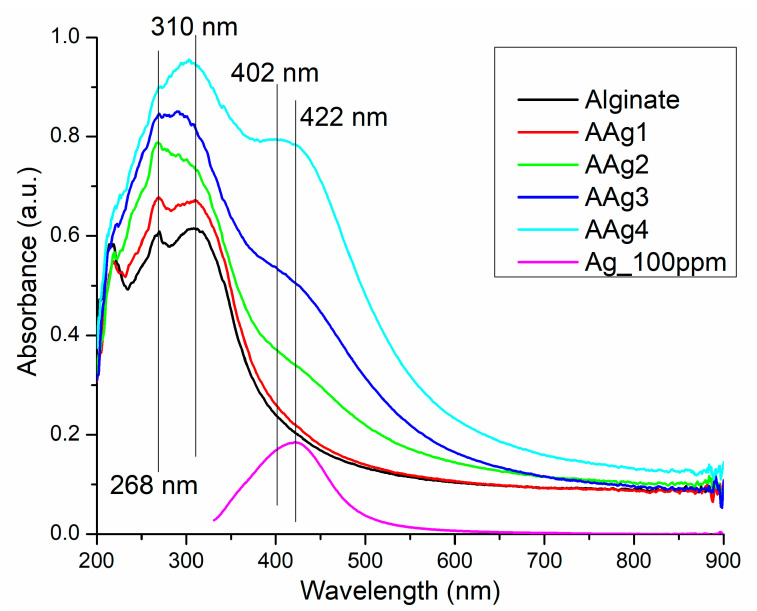
UV-Vis spectra for Ag NPs, alginate, and AAg1–AAg4 films.

Our Ag NPs solution was proven stable over 3 months’ storage, at room temperature, under dark conditions, the spectrum being virtually unchanged, with the band exhibiting the maximum absorption peak at 422 nm (Figure 5). This indicates that the Ag NPs are well capped by the PVP and do not agglomerate [73]. The peak broadness indicates that, although small, the size of nanoparticles varies, as seen also from TEM images, from 5 to 25 nm.

The UV-Vis spectra for the alginate and AAg1–AAg4 films, presented in Figure 5, indicates the existence of a darkening effect in correlation with the increase in Ag NPs content. The AAg1 film has a marginally higher absorbance in UV region and is virtually identical with alginate film in the visible domain. For the AAg2–AAg4, the increased absorbance can be observed in the violet-blue region, which starts as a shoulder in the AAg2 sample, and develops into a separate broad band with the peak at 402 nm for AAg4. The presence of the 402 nm band can be assigned to the increasing concentration of Ag NPs (ten times higher in AAg4 than in AAg1).

The UV absorbance is also increasing with the Ag NPs content. Both UV and Vis light barriers are important features for food packaging, as a high absorbance will protect the critical nutrients (vitamins, lipids, or proteins) from photo-oxidation reactions promoted by high-energy photons. Different superscripts (a, b) in the last column are significantly different (*p* < 0.05). Values are given as the mean ± SD from triplicate determination.

The incorporation of Ag NPs into the alginate film is increasing the opacity, but only marginally (Table 3), from 0.48 ± 0.02 to a maximum of 0.67 ± 0.04, and therefore the alginate films can be considered transparent [74].

**Figure 6:**
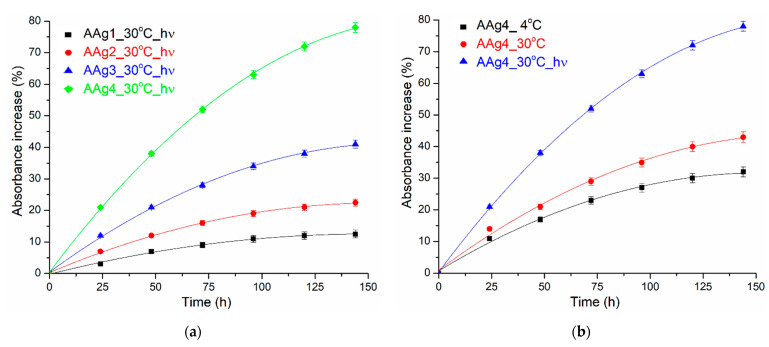
Color darkening (absorbance increase at 402 nm) for AAg1–AAg4 films: as function of Ag NPs content (**a**) and as function of temperature and light presence (**b**).

The AAg1–AAg4 films can become less transparent over time, depending on the storage conditions, e.g., temperature or light presence. As expected, a higher Ag NPs content will promote a large absorbance change (Figure 6a). Temperature change (4 °C vs. 30 °C) will also increase the absorbance change, but the presence of light has the highest impact on the film darkening (Figure 6b). The main reason for the changing of color is the oxidation of Ag NPs [75]. As the reaction rate will increase with the temperature, the samples stored at 30 °C will darken quicker than those stored at 4 °C. Similar reports are found in the literature [76,77]. This indicates that such films can be used as a time-temperature indicator (TTI) for storage conditions of food. Cumulative higher temperature and light (which for shelf food translate into exposure time) lead to a change of transparency, and therefore can indicate the freshness of packed food.

**Figure 7:**
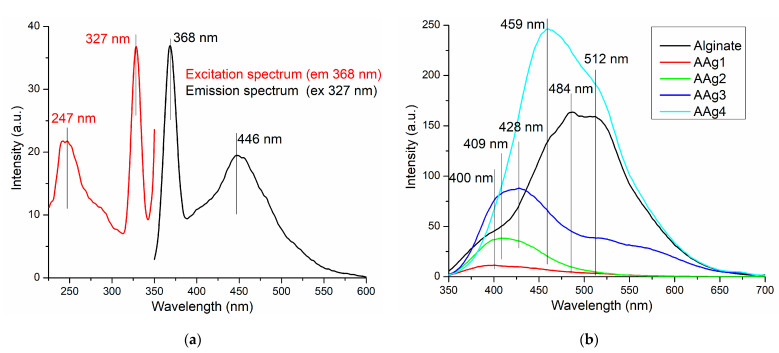
The fluorescence spectra for Ag NPs (**a**) alginate and AAg1–AAg4 films (**b**).

The Ag NPs are well known for their fluorescent properties. The literature reports different emission peaks in dependence on the excitation wavelength and Ag NPs morphology [78]. Usually, it is reported that the surface plasmon extinction peak is around 400 nm, while the fluorescent emissions at higher wavelengths are attributed to ultra-small Ag NPs (~5 nm) [79]. Here, we report Ag NPs that present the surface plasmon extinction peak at 368 nm, with a second emission band at 446 nm (Figure 7a). Surface plasmon related emission peaks under 400 nm have been reported in the previous literature, e.g., 384 nm [80], 375 nm [81], or 330 nm [78]. The broad visible emission band from 446 nm can be attributed to *sp → d* radiative transitions due to Ag–Ag interactions. Similar shaped spectra, with surface plasmon emission placed in UV region accompanied by a broad visible emission peak in blue region are reported [78,82].

The fluorescence spectra for the alginate and AAg1–AAg4 films presented in Figure 7b, indicates the existence of Ag NPs–alginate interactions. The addition of a small quantity of Ag NPs in AAg1 sample leads to an emission band centered on 400 nm, much weaker than those corresponding to the Ag NPs or alginate. The interactions between Ag NPs and alginate polymeric chains are probably responsible for blocking the surface plasmon emission, but also for quenching the alginate fluorescence. Increasing the Ag NPs content of the composite films is generating a stronger and broader visible emission band, while the peaks are red-shifted towards 459 nm, with a shoulder at 512 nm.

This can be attributed to the existence of local Ag NPs agglomerations (as seen in ESEM images), which promotes Ag–Ag interactions, either directly or mediated by moieties of alginate. A similar effect of nanoparticles on alginate fluorescence was reported in [4].

### 3.5. Thermal Analysis TG/DSC–FTIR

**Figure 8:**
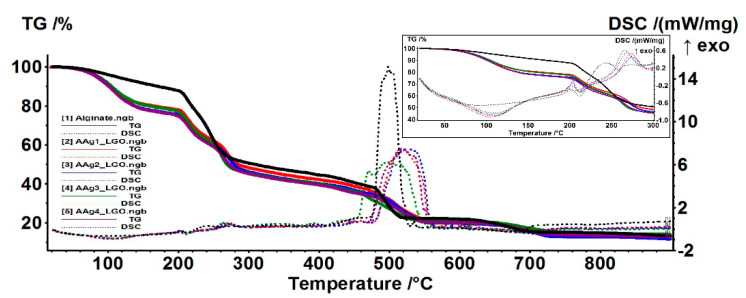
TG/DSC curves for alginate and AAg1–AAg4 films.

The thermal analysis of alginate and AAg1–AAg4 samples indicates that the composite films have a lower thermal stability than the simple alginate one, the mass loss starting at low temperatures, i.e., under 100 °C (Figure 8).

The alginate film starts losing mass at about 70 °C, most probably water, the process being accompanied on DSC curve by a weak endothermic effect with minimum at 93.2 °C. Up to 200 °C the sample is losing 12.14% of its initial mass.

**Figure 9:**
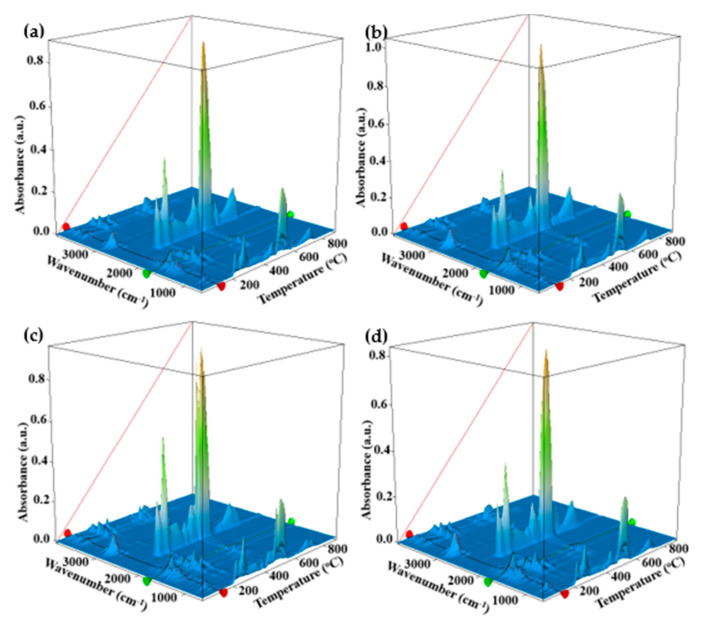
The 3D FTIR plot of evolved gases for AAg1 (**a**); AAg2 (**b**); AAg3 (**c**); and AAg4 (**d**).

The samples AAg1–AAg4 present a larger mass loss up to 200 °C (22–25%), the corresponding endothermic effect being more intense and shifted towards higher temperatures (105–110 °C). This can be attributed to the elimination of various volatile compounds belonging to LGO incorporated into the alginate films, which means that approximatively 10% of the sample mass is consisting of LGO. The hypothesis is sustained by the FTIR analysis of evolved gases. Figure 9, FTIR 3D plot vs. T (°C), reveals at low temperatures the presence of absorption bands around 3000 cm^−1^ which are attributed to C-H vibration in various hydrocarbons, which can be attributed to elimination of LGO volatile components (Figure 9).

The oxidation process of alginate matrix starts after 200 °C, when multiple exothermic effects, with low intensity, can be observed on DSC curve (inset of Figure 8). The FTIR profile for the absorption band from 2355 cm^−1^, corresponding to CO_2_ elimination from sample (Figure 9), presents multiple peaks after 200 °C. Each peak corresponds to an oxidation process, the most intense being attributed to the burning of residual carbonaceous mass around 500 °C.

### 3.6. Water Vapor Permeability (WVP)

The barrier properties of alginate films are very important, as they must prevent the loss of flavor, water, or other volatile substances from the packed food [83]. The water vapor permeability (WVP) values best describe the moisture capacity to migrate between environment and food, through the packaging film. Microbial spoilage can be related to good moisture permeability. Therefore, it is important to determine the values for WVP (Table 4).

The simple alginate film presented an average value for WVP when compared to similar reports [30,83]. The higher value can be explained by the glycerol addition, which generates hydrogen bonds with alginate and therefore increases the inter-chains distance, allowing moisture to penetrate easier [84].

Smaller values for WVP are obtained for the AAg1–AAg4 films, but they are not statistically different among them. This can indicate that the main cause for this decrease is the presence of LGO rather than the addition of Ag NPs. The clear hydrophobic nature of LGO generates micron and sub-micron drops inside the alginate films.

As thermal analysis data indicates, a large proportion of LGO is trapped inside the polymeric matrix (~10% *w*/*w*).

**Figure 10:**
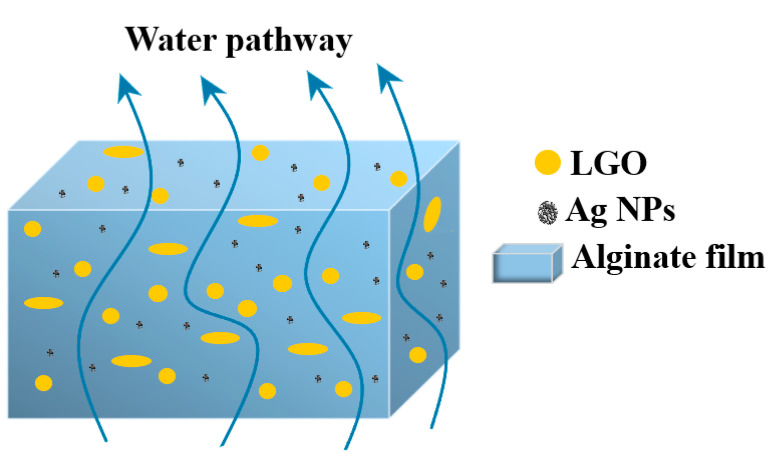
The proposed mechanism behind decrease in WVP values for AAg1–AAg4 films.

As such, the water molecules pathway becomes longer and obstructed by hydrophobic zones represented by LGO [85], which act as physical barriers [13,83] (Figure 10).

### 3.7. Swelling Study

The swelling behavior was assessed in PBS (pH = 7.4) and in distilled water. An increase in the swelling capacity with the increase in Ag NPs content can be observed in both cases (Table 5).

As the Ag NPs amount increased, the AAg1–Aag4 films have a higher capacity of water uptake. This increasing capacity can be related to the pores’ dimensions at the microscopic level, and with the space between alginate chains at molecular level. The Ag NPs are stabilized with PVP. The presence of more silver nanoparticles and PVP can induce a wider gap between alginate chains, and therefore a higher water retention capacity. The pores can act as traps for water molecules. A higher pore density therefore can trap a larger water quantity (e.g., for AAg4 film).

In water, the films were swelling close to maximum capacity in 2 h, with more than half of the water quantity being retained in first 15 min. The swelling increased slowly up to 3 h, further measurements indicating a weight stabilization (the measurement at 24 h is presented in Table 5, but the films were stable for 30 days).

The swelling study in PBS produced larger values for water retention capacity (up to six times higher than those obtained for water study).

This can be explained by the slow replacement of Ca^2+^ ions with Na^+^ ones, which leads to the destruction of the Ca-alginate “egg-box” structure. This leads to an increasing gap between polymeric chains and, as such, larger amounts of water can be retained. As the replacement of Ca^2+^ ions proceeds, the resulting sodium alginate will start to dissolve, therefore the sample will start to lose mass. At 72 h the films are completely disintegrated in PBS solution.

### 3.8. Antibacterial Activity

**Figure 11:**
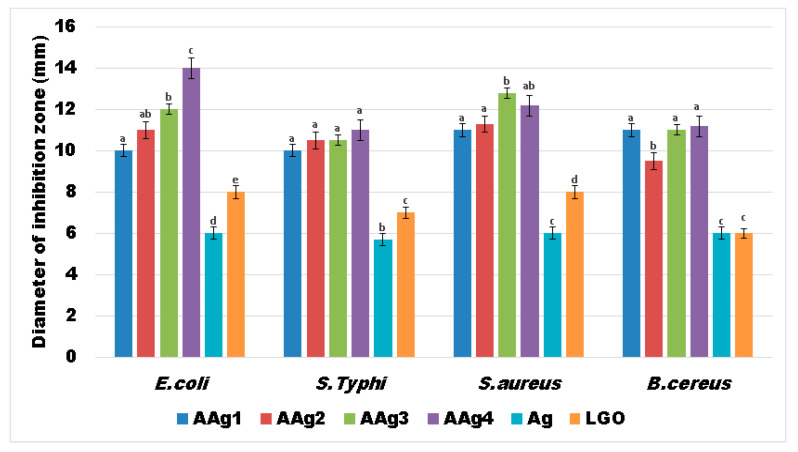
Measured growth inhibition diameters (mm) for the evaluated bacterial strains in the presence of AAg1–AAg4 films. Different small letters indicate statistically significant differences between films (*p* < 0.05).

The results obtained for the AAg1–AAg4 films against four relevant food born infections bacterial strains, two Gram-positive (*S. aureus*; *B. cereus*) and two Gram-negative (*S.* Typhi; *E. coli*), indicate a strong antibacterial activity (Figure 11).

This suggests a large spectrum of antibacterial activity for the alginate films that contains both Ag NPs and LGO. As the simple alginate film exhibited no antibacterial activity, it is safe to assume that the strong antibacterial effect of AAg1–AAg4 films comes from Ag NPs combined with substances from the essential oil.

The results obtained suggest that growth inhibition is dependent of Ag NPs concentration in case of *E. coli* and *S. aureus*, the largest inhibition zones being observed for AAg3 and AAg4 samples, followed by AAg2 and AAg1 (Figure 11). In the case of *S.* Typhi and *B. cereus* the diameter of inhibition zone does not differ significantly among AAg1–AAg4 samples, but it has a consistently constant high value. This indicates that both Ag NPs and LGO act synergically, conferring a strong antibacterial activity.

LGO alone has a lower antibacterial effect, but in combination with Ag NPs the values for growth inhibition diameters are higher. The Ag NPs solution also presented a modest, constant value for the growth inhibition zone among all four tested strains. Therefore, the high values obtained for the antibacterial activity of AAg1–AAg4 suggest the existence of synergism between Ag NPs and LGO. The probable mechanism is related to the LGO changing the adenosine triphosphate concentration and hyperpolarization of the cell wall, and decreasing of the cytoplasmic pH [86,87], which in turn makes it easier for the silver ions released by the Ag NPs to damage the membrane and bind the proteins and enzymes [88], disrupting vital processes inside the cell.

**Figure 12:**
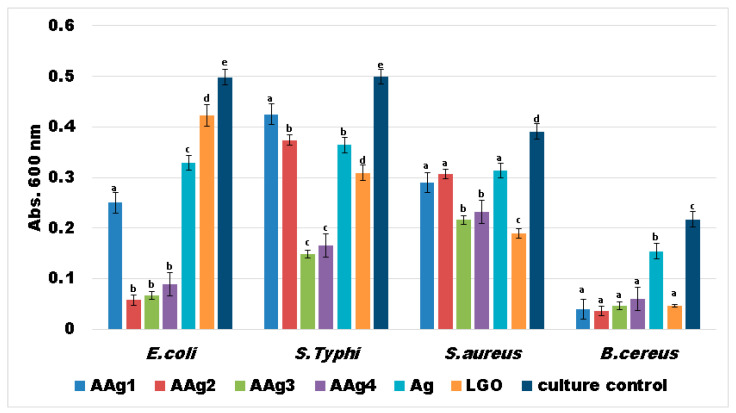
Absorbances at 600 nm indicating growth of planktonic cultures in the presence of AAg1–AAg4 films for 24 h at 37 °C. Different small letters indicate statistically significant differences between films (*p* < 0.05).

Strong planktonic growth inhibition was observed in cases of *E. coli* and *B. cereus* (for AAg2–AAg4 films especially) indicating that the antibacterial compounds from LGO and Ag NPs could be released from the alginate polymeric matrix and affect the evolution of free bacterial cells (Figure 12). In cases of *S.* Typhi and *S. aureus* strains, the development of individual cells was also inhibited mostly by AAg3–AAg4 samples, suggesting a susceptibility to the presence of larger amounts of Ag NPs.

**Figure 13:**
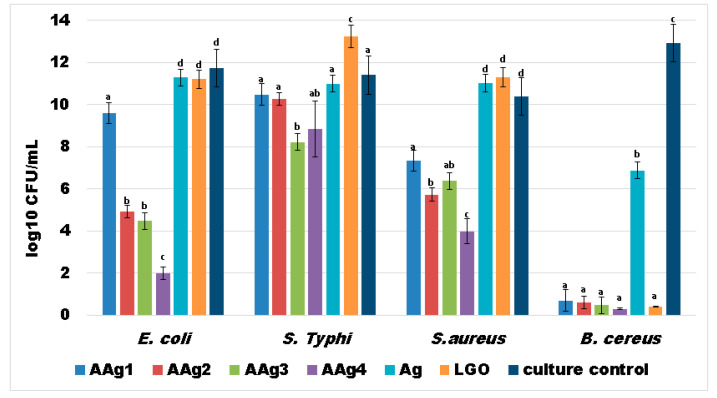
Values of log_10_CFU/mL for the tested bacterial strains, expressing biofilm embedded cells developed on control and AAg1–AAg4 films after 24h incubation. Different small letters indicate statistically significant differences between films (*p* < 0.05).

Similar results were also obtained for the bacterial biofilm development and its attachment in the presence of AAg1–AAg4 films (Figure 13). The obtained data indicate that biofilm development is significantly reduced in the cases of *E. coli* and *B. cereus*. The *B. cereus* was again the most susceptible strain, as we saw in the case of planktonic growth. In the case of *E. coli* and *S. aureus,* a clear dependence of Ag NPs is evidenced, consistent with the results from growth inhibition diameter measurements.

Biofilm growth inhibition results, presented in Figure 13, indicate that the films are highly efficient towards Gram-positive *B. cereus* strain. A good antibacterial activity can also be observed for the other strains, especially for AAg4 sample. The samples with higher Ag NPs content exhibited best antibacterial activity, so we can state that biofilm growth inhibition is dependent of Ag NPs concentration. The results suggest that these films can be tailored to combat specific pathogens, depending on the desired application and susceptibility of microbial strains.

The literature indicates that Ag NPs present a stronger antibacterial activity against *S. aureus* than *E. coli* [89,90]. For the LGO, as well as the antifungal activity, a strong antibacterial activity against Gram-positive bacterial strains such as *B. cereus* is reported [16,91].

Our study has revealed that combining the two antibacterial agents, Ag NPs and LGO, a strong synergic antibacterial activity is obtained. The AAg1–AAg4 films are efficient against both types of pathogens, Gram-positive and Gram-negative, despite the former being usually more resistant due to its complex cellular wall [91,92].

### 3.9. Evaluation of Potential Use of AAg1–AAg4 Films as Food Packaging

**Figure 14:**
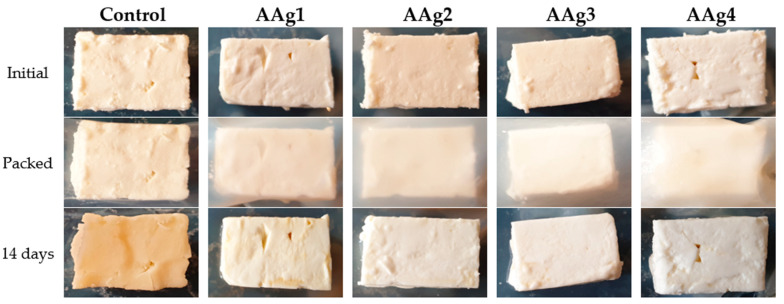
Visual appearance of soft cheese bits packaged in alginate control film and AAg1–AAg4 films, initial and after 14 days storage at 4 °C and 75% relative humidity.

Cheese is an important food made from casein, fat, and water. When it is not salted, uncontrolled and extensive development of microorganisms is contributing to cheese’s short shelf life. Different cheese varieties need some bacterial cultures to be produced, and this creates an infection hazard from the cheese-borne species such as *Escherichia coli*, *Salmonella enterica*, or *Staphylococcus aureus* [4]. Samples of soft cheese of approximatively 10.00–13.00 g were weighed and packed in alginate or AAg1–Aag4 films. The samples were stored for 14 days at 4 °C ± 1 °C and 75% RH. After 14 days, the samples were visually checked, weighed, and the pH was measured. The cheese bits appearances (Figure 14), mass loss (Table 6), and pH data suggest that AAg1–AAg4 films can preserve cheese for up to 2 weeks.

The obtained results indicate that the cheese samples stored in AAg1–AAg4 films were better preserved comparatively with the control sample. The white, soft texture was maintained for samples packed in AAg1–AAg4 films, while the control sample changed its color and became harder. The control sample turned dark yellow due to spoilage microorganisms’ growth. The sample packed in AAg1 film also presented some minor spoilage coloration on the edge, but the samples packed in AAg2–AAg4 films retained the initial aspect, color, surface texture, and softness. This indicates that these antimicrobial films are capable of preserving the soft cheese and extending the shelf life.

The weight of the cheese samples was measured at the start and after 14 days of storage (Table 6). The samples packed in AAg1–AAg4 films presented a small mass loss, around 2.50% value, while the control sample lost 39.32%. This can be explained by the better barrier properties of the composite films, as seen in Section 3.6. The cheese eliminates water vapors, and with less hindrance from the simple alginate film, they are lost in surrounding environment.

Modification of the pH value for the cheese can indicate the presence of spoilage microorganisms. Therefore, the pH value was measured initially and after 14 days. For the samples packed in AAg1–AAg4 films, the value remained constant at 4.5. Similar constant values are reported in the literature when spoilage is absent [93]. For the control sample, the pH value dropped to 4.3, most probably due to growth and fermentation activity of microorganisms.

Nevertheless, a full study on the migration magnitude of Ag NPs to the food surface to assess the possible toxic effects is mandatory.

## 4. Conclusions

Novel innovative biodegradable alginate films were obtained and characterized. A strong antibacterial activity was conferred by the addition of Ag NPs and lemongrass essential oil. The antimicrobial agents are shown to act in a synergic fashion, the AAg1–AAg4 samples presenting a large antibacterial activity spectrum, against both Gram-positive and Gram-negative strains, best results being obtained against *B. cereus*. The antibacterial films were tested as packaging for soft cheese, the preliminary test indicating a good capacity to preserve it up to 14 days. The light and water barrier properties of the alginate films were enhanced by the addition of LGO and Ag NPs. The films are transparent, but at 30 °C and in the presence of light they tend to darken, with the absorbance increasing by up to 78% compared with those stored at 4 °C in the absence of light, where the absorbance increased only with max 32%, in a six day interval. This process can be used to monitor the storage conditions for sensitive food.

## Figures and Tables

**Table 1:** The alginate–Ag NPs–lemongrass essential oil (LGO) films composition.

Sample Code	Alginate (g in 100 mL Water)	Ag NPs (mL of 100 ppm Solution)	Glycerol (mL Solution)	LGO (mL)
A	3.00	0	2	0
AAg1	3.00	5.0	2	1.0
AAg2	3.00	10.0	2	1.0
AAg3	3.00	25.0	2	1.0
AAg4	3.00	50.0	2	1.0

**Table 2:** Assignment of relevant IR absorption bands of alginate (A) and AAg1–AAg4 films.

Sample/Assignment	A	AAg1	AAg2	AAg3	AAg4
υ_as_C-O-C	1031	1028	1027	1026	1027
υ_s_COO^-^	1416	1408	1407	1409	1410
υ_as_COO^-^	1596	1602	1600	1602	1604
C = O group of LGO [67]		1740	1738	1738	1739
υC-H (sat)	2921	2930	2925	2921	2921
υO-H	3278	3277	3270	3284	3287

**Table 3:** Thickness and opacity for alginate (A) and alginate/Ag NPs/LGO (AAg1–AAg4) films.

Sample	Alginate	AAg1	AAg2	AAg3	AAg4
Thickness (mm)	0.22 ± 0.01	0.21 ± 0.02	0.27 ± 0.02	0.30 ± 0.03	0.35 ± 0.02
Opacity	0.48 ± 0.02 ^a^	0.51 ± 0.05 ^a^	0.54 ± 0.04 ^a^	0.55 ± 0.06 ^a^	0.67 ± 0.04 ^b^

Different superscript letters indicate statistically significant differences between films (*p* < 0.05).

**Table 4:** Water vapor permeability for simple alginate and AAg1–AAg4 films.

Film Code	WVP (10^−10^ g/Pa∙m∙s)
A	5.718 ± 0.011 ^a^
AAg1	2.753 ± 0.042 ^b^
AAg2	2.706 ± 0.035 ^b^
AAg3	2.696 ± 0.024 ^b^
AAg4	2.691 ± 0.054 ^b^

Different superscript letters indicate statistically significant differences between films (*p* < 0.05).

**Table 5:** Swelling capacity (as mass increase in %) for the alginate and AAg1–AAg4 films.

Sample	Water PBS					
	0.25 h	0.5 h	1 h	2 h	3 h	24 h
A	42.54%	61.22%	86.14%	101.83%	104.69%	102.76%
	81.99%	182.05%	374.36%	595.63%	659.28%	741.75%
AAg1	61.12%	84.92%	103.67%	108.38%	105.53%	100.43%
	113.94%	276.24%	451.33%	556.02%	596.58%	564.89%
AAg2	82.97%	95.59%	108.65%	114.35%	119.33%	121.06%
	167.42%	347.91%	489.04%	589.77%	623.66%	704.31%
AAg3	110.13%	123.90%	130.79%	126.41%	128.91%	128.53%
	231.88%	454.65%	580.89%	647.52%	691.74%	675.24%
AAg4	136.47%	151.97%	162.99%	167.90%	169.77%	168.81%
	295.55%	549.36%	696.36%	719.23%	857.85%	799.79%

**Table 6:** Weight loss for cheese bits coated with alginate control and AAg1–AAg4 films during storage.

Sample/ Weight Loss (%)	A	AAg1	AAg2	AAg3	AAg4
14 days	39.32 ± 0.51 ^a^	2.39 ± 0.21 ^b^	2.31 ± 0.17 ^b^	2.61 ± 0.26 ^b^	2.55 ± 0.24 ^b^

Different superscript letters in the same column indicate statistically significant differences between films (*p* < 0.05).

## Data Availability

The data is included in the main text and/or the supplementary materials.

## Supplementary Materials

The following are available online at https://www.mdpi.com/article/10.3390/nano11092377/s1, Materials and Methods. Figure S1: Preparation scheme for the alginate films with Ag NPs and LGO. Figure S2: XRD of obtained Ag NPs (JCPDS 04-0783) (a) and of AAg4 film (b). Figure S3: TG / DSC curves for alginate (a); AAg1 (b); AAg2 (c); AAg3 (d) and AAg4 (e) films.
