# Supplementary material for: Antibacterial Biodegradable Films Based on Alginate with Silver Nanoparticles and Lemongrass Essential Oil–Innovative Packaging for Cheese
Source: Nanomaterials (Basel). 2021 Sep 13;11(9):2377. doi: 10.3390/nano11092377 (PMC8467694; doi:10.3390/nano11092377)
Supplement: Supplementary file 1 [file nanomaterials-11-02377-s001.zip › nanomaterials-1362391-supplementary.pdf]

nanomaterials

MDPI

Antibacterial Biodegradable Films Based On Alginate with Silver Nanoparticles and Lemongrass Essential Oil–Innovative Packaging for Cheese

Ludmila Motelica [1], Denisa Ficai [1], Ovidiu-Cristian Oprea [1,*], Anton Ficai [1,2], Vladimir-Lucian Ene [1], Bogdan-Stefan Vasile [1], Ecaterina Andronescu [1,2] and Alina-Maria Holban [1,3]

[1] Faculty of Applied Chemistry and Material Science, University Politehnica of Bucharest, 060042 Bucharest, Romania; motel ica_ludmila@yahoo.com (L.M.); denisa.ficai@upb.ro (D.F.); anton.ficai@upb.ro (A.F.); vladimir.l.ene@gmail.com (V.-L.E.); vasile_bogdan_stefan@yahoo.com (B.-S.V.); ecaterina.andronescu@upb.ro (E.A); alina.m.holban@bio.unibuc.ro (A.M.H.)
[2] Academy of Romanian Scientists, 050045 Bucharest, Romania
[3] Microbiology & Immunology Department, Faculty of Biology, University of Bucharest, 077206 Bucharest, Romania
* Correspondence: ovidiu.oprea@upb.ro or ovidiu73@yahoo.com; Tel.: +40-214-023-986

2. Materials and Methods

2.4. Characterization of Alginate Films

2.4.1. Microstructural Analysis

In order to investigate the films surface morphology and microstructure scanning electron micrographs were obtained using an environmental scanning electron microscope VERSA 3D (ESEM, Thermo-Fisher, former FEI Company, Eindhoven, The Netherlands) that allows the adjustment of pressure, temperature and humidity inside the work chamber. A secondary electron detector in gaseous conditions, along with a low vacuum / ambient conditions limiter were used for image acquisition. The sample was mounted on a Peltier-effect assisted cooling support and the generated heat was dissipated by a continuous flow of water at 16 °C.

Bright Field and High Resolution a Transmission Electron Microscopy (BF-TEM, HR-TEM) images coupled with Selected Area Electron Diffraction (SAED) pattern were recorded using High-Resolution 80-200 TITAN THEMIS transmission microscope equipped with an Image Corrector and 4 EDXS detector in the column, purchased from the FEI (Oregon, United States). 0.1 mL from the Ag NPs solution was dispersed into deionized water, left to ultrasonicate for 15 minutes and after that 10 μL of the suspension was placed on a 400 mesh lacey carbon-coated copper grid at room temperature and analyzed. The microscope operates in transmission mode at a 200 kV voltage.

2.4.2. Fourier Transform Infrared Spectroscopy

By using Fourier transform infrared spectroscopy (FTIR) in the wavenumber range 4000–400 cm^{-1} we investigated the presence of certain functional groups and possible interactions between components of the alginate films. A Nicolet iS50 FTIR spectrometer (Thermo Fisher Scientific Inc., Waltham, MA, USA), equipped with a DTGS detector, was used to record the spectra. We used a resolution of 4 cm^{-1}, and each spectra was obtained by averaging 32 scans.

FTIR 2D maps were recorded with a Nicolet iS50R FTIR microscope (Thermo Fisher Scientific Inc., Waltham, MA, USA), with DTGS detector, in the wavenumber range 4000–600 cm^{-1}. The 2D FTIR maps were used to obtain information about the spatial distribution of the components.

2.4.3. Photoluminescence Spectroscopy

A Perkin Elmer (Waltham, MA, USA) LS55 spectrometer was used to measure the photoluminescence spectrum (PL). A Xe lamp was used as a UV light source at ambient temperature, the fluorescence being measured in the range 350–800 nm. The spectra were recorded with a scan speed of 200 nm min^{-1}, excitation and emission slits of 10 nm, and a 1% attenuator, due to high emission intensity that exceeded the instrument limit. An excitation wavelength of 320 nm was used.

2.4.4. UV-Vis Spectroscopy

A JASCO V560 spectrophotometer (JASCO Inc., Easton, PA, USA) was used to measure the UV–Vis spectra. The device was equipped with a 60 mm integrating sphere (ISV-469) and a film holder for the samples. The spectra were recorded with a speed of 200 nm min^{-1}, in the domain 200–900 nm.

The opacity values were calculated as $A_{600}/x = -\log T_{600}/x$, where A_{600} is the absorbance at 600 nm, T_{600} is the fractional transmittance at 600 nm and x is the film thickness in mm. A higher opacity value indicates that the film is less transparent [59].

2.4.5. Thermal Analysis

Thermal analysis, TG-DSC (thermogravimetry and differential scanning calorimetry), was performed with a STA 449C F3 apparatus, from Netzsch (Selb, Germany). Each sample weighed approximatively 10 mg. The samples were placed in an open alumina crucible and heated up to 900 °C with 10 $K\cdot min^{-1}$ rate, under flow of 50 $mL\cdot min^{-1}$ dried air. As reference, we used an empty alumina crucible. The evolved gases were analyzed with a FTIR Tensor 27 from Bruker (Bruker Co., Ettlingen, Germany), equipped with a thermostated gas cell.

2.4.6. Water Vapor Permeability (WVP)

For the determination of water vapor permeability (WVP) we used permeation cups with a diameter of 30 mm, sealed with a sample film, as described in [60]. In each cup we placed 1 g of dried $CaCl_2$. The permeation cups were placed in a container at a temperature of 25 °C and 100% relative humidity. Their weight was measured at fix intervals (8 h) for four days.

2.4.7. Swelling Capacity

The swelling capacity was determined as described in [61]. Shortly, square samples of ~ 3 × 3 cm were cut from the fresh films and were dried in a desiccator for 48 h. Once dried, the samples were weighed (± 0.2 mg) (W_0), then placed in 200 mL water or phosphate buffer saline (PBS) to allow swelling. The samples were weighted at each 15 min first, then at each 30 min for three hours, and finally at 24 h intervals for next days as the maximum swelling capacities were attained. The equation 1 formula for degree of swelling (D) was used to calculate the swelling ratio:

$$D = (W_t - W_0)/W_0 \quad (1)$$

2.5. Antibacterial Assay

The antibacterial activity was evaluated against two model Gram-positive (*Bacillus cereus* ATCC 13061 and *Staphylococcus aureus* ATCC 25923) and two Gram-negative (*Escherichia coli* ATCC 25922 and *Salmonella enterica* Typhi ATCC 14023) bacteria, which are relevant in food bacterial contamination. The strains were maintained as glycerol stocks at −80 °C. All experiments were designed and performed in triplicate.

2.5.1. Antibacterial Qualitative Assessment—Growth Inhibition

To qualitatively screen the antibacterial effect of the obtained materials, we utilized an adapted diffusion assay, respecting the general rules exposed in the CLSI 2020 and in

our recent study [62]. The obtained 0.5 McFarland bacterial suspensions (1.5 × 10^8 CFU/mL), previously prepared in sterile saline (0.9% NaCl solution) were utilized as a standardized inoculum to swab inoculate Petri dishes containing nutritive agar. From the obtained films, 6 mm diameter discs were cut. The discs were sterilized by UV exposure for 30 min before use. Sample discs were placed in aseptically conditions on the inoculated Petri-dishes and these were incubated for 20 h at 37 °C. After incubation, a diameter of growth inhibition developed around each disc specimen. This diameter was measured and expressed in mm.

2.5.2. Evaluation of the Planktonic Development of Microorganisms

Planktonic growth in the presence of the obtained materials was analyzed in nutritive broth. Discs of 6 mm in diameter were placed in sterile 24-well plates. Then, 1 mL of nutritive broth and 10 µL of the previously obtained 0.5 McFarland bacterial suspensions in PBS were added. Specimens were allowed to incubate for 24 h at 37 °C. To spectrophotometrically evaluate the growth of planktonic (free-floating) cultures, 150 µL of the obtained bacterial culture were transferred to 96-well plates and the absorbance at 600 nm was measured.

2.5.3. Monospecific Biofilm Development

The antibiofilm efficiency was determined by transferring sterile sample discs with 6 mm in diameter in sterile 24-well plates containing 1 mL nutritive broth, followed by the inoculation of 10 µL of bacterial suspension with standard density of 0.5 McFarland. The as prepared plates were incubated for 24 h at 37 °C. The samples were carefully washed afterwards with 1 mL solution of sterile saline. In the end, the samples were transferred in 1.5 mL centrifuge tubes, in 1000 µL solution of sterile saline. The as obtained samples were then vortexed for 30 s to ensure the detachment of biofilm cells in suspension. After this, serial 10 fold dilutions were obtained and were used to inoculate the nutrient agar in order to evaluate the viable colony formation, expressed as log_{10}CFU (colony forming units)/mL.

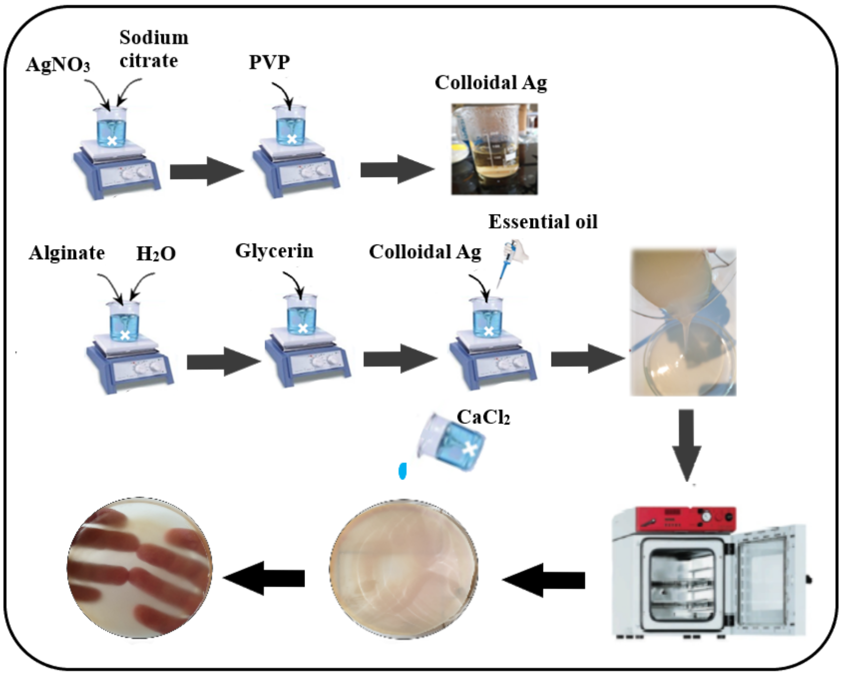

Figure S1. S. Preparation scheme for the alginate films with Ag NPs and LGO.

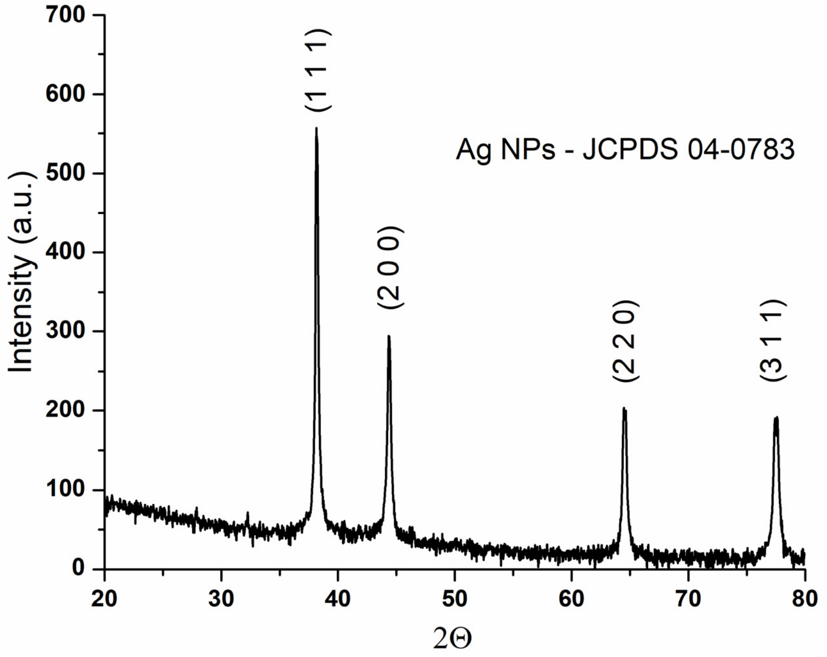

(a)

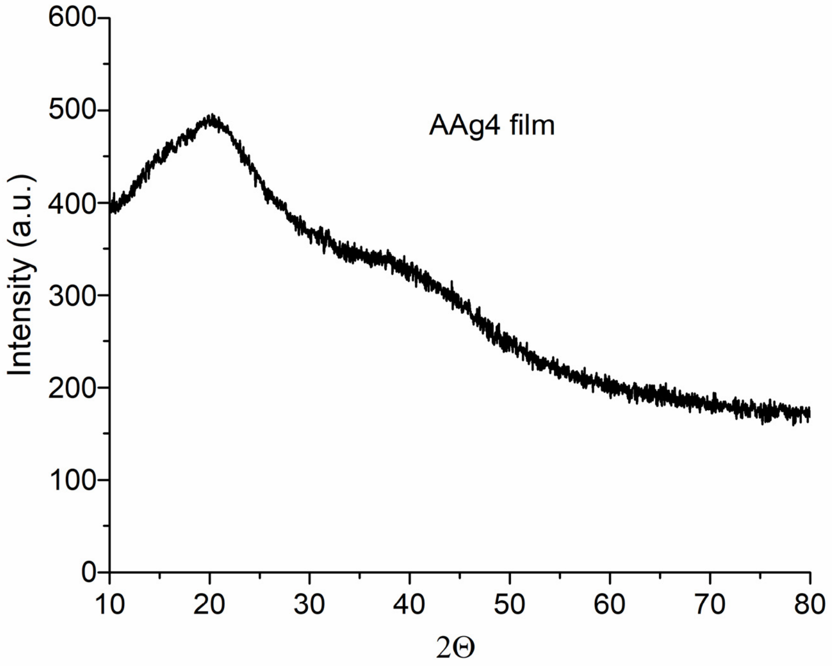

(b)

Figure S2. S. XRD of obtained Ag NPs (JCPDS 04-0783) (**a**) and of AAg4 film (**b**).

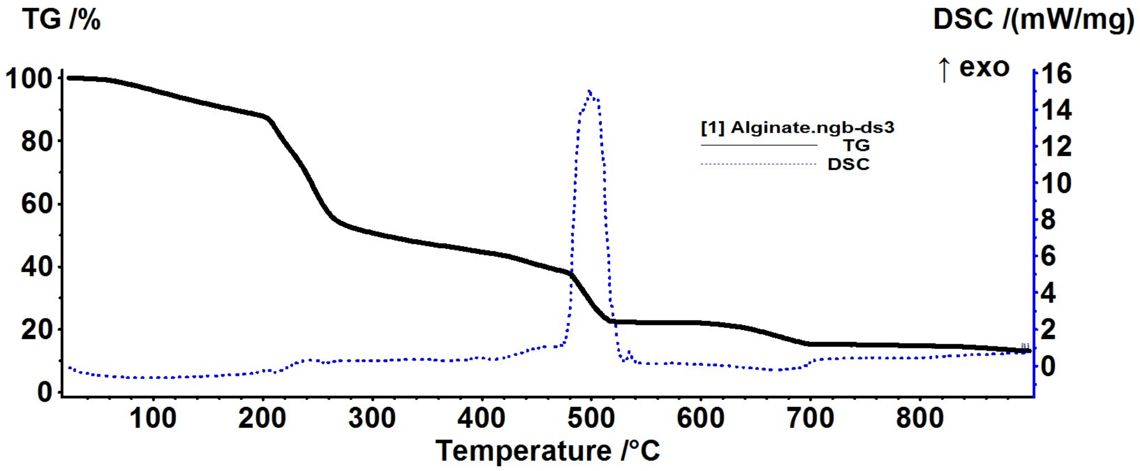
TG /%
DSC /(mW/mg)
↑ exo
[1] Alginate.ngb-ds3
TG
DSC
Temperature /°C

(a)

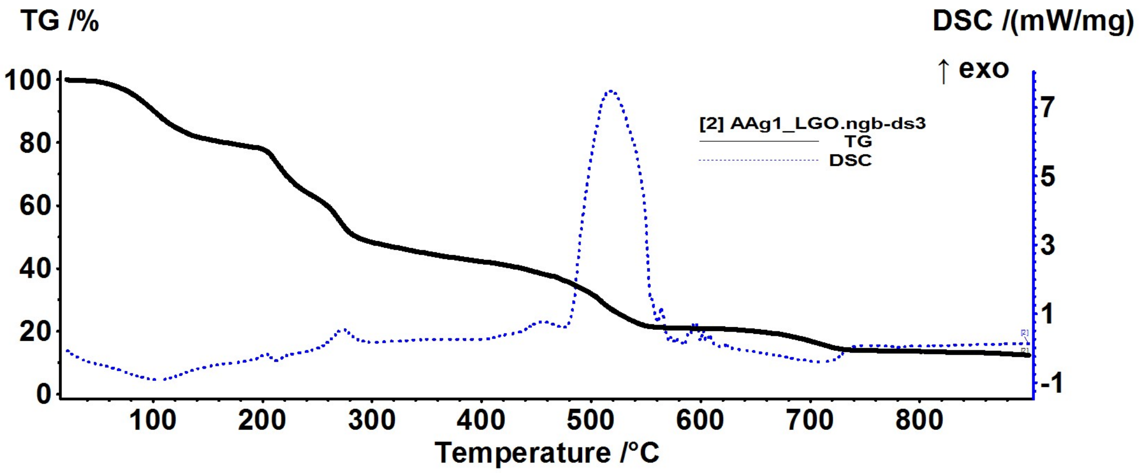
TG /%
DSC /(mW/mg)
↑ exo
[2] AAg1_LGO.ngb-ds3
TG
DSC
Temperature /°C

(b)

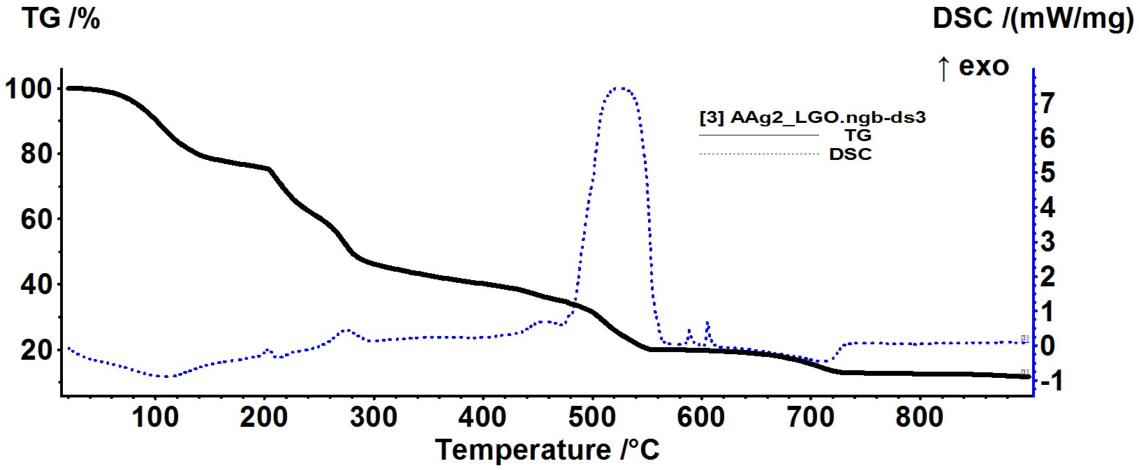
TG /%
DSC /(mW/mg)
↑ exo
[3] AAg2_LGO.ngb-ds3
TG
DSC
Temperature /°C

(c)

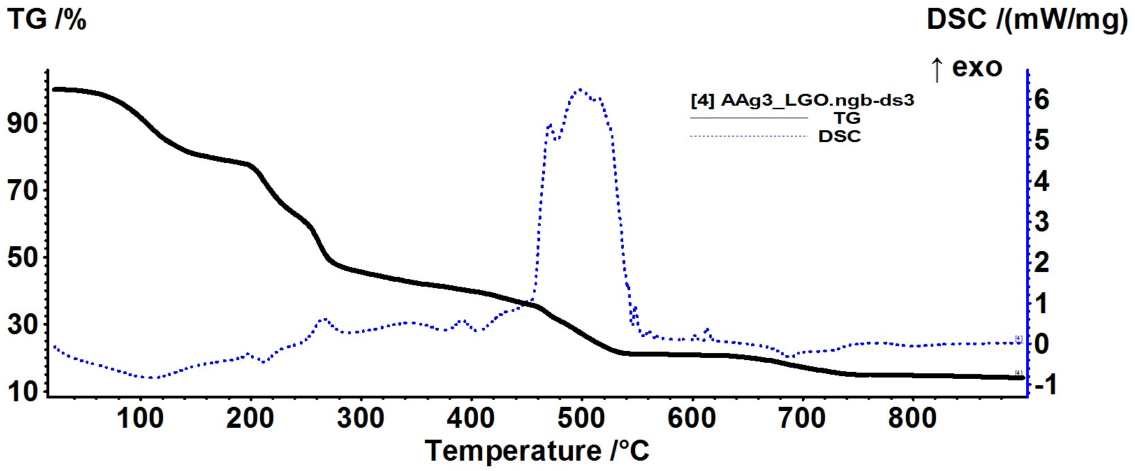

(d)

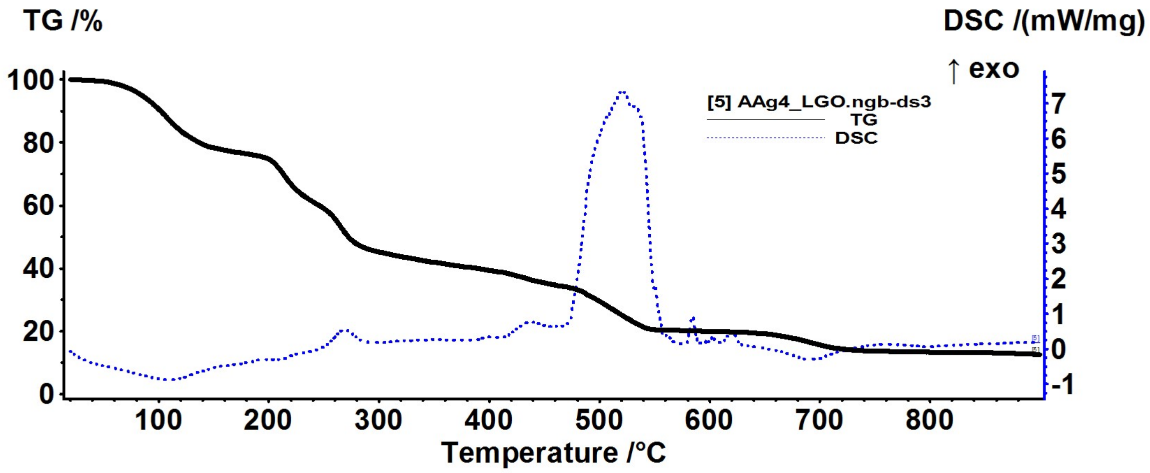

(e)

Figure S3. S. TG / DSC curves for alginate (**a**); AAg1 (**b**); AAg2 (**c**); AAg3 (**d**) and AAg4 (**e**) films.
